# Downregulation of TAP1 and TAP2 in early stage breast cancer

**DOI:** 10.1371/journal.pone.0187323

**Published:** 2017-11-01

**Authors:** Andrea M. Henle, Aziza Nassar, Danell Puglisi-Knutson, Bahaaeldin Youssef, Keith L. Knutson

**Affiliations:** 1 Department of Biology, Carthage College, Kenosha, Wisconsin, United States of America; 2 Department of Laboratory Medicine and Pathology, Mayo Clinic, Rochester, Minnesota, United States of America; 3 Department of Immunology, Mayo Clinic, Jacksonville, Florida, United States of America; University of South Alabama Mitchell Cancer Institute, UNITED STATES

## Abstract

TAP1-TAP2 heterodimeric complexes are recognized as the transporter associated with antigen processing of major histocompatibility complex class I peptides for recognition by tumor-specific cytotoxic T lymphocytes. In this study, we investigated the immunohistochemical expression of TAP1 and TAP2 in 160 patients with breast cancer and correlated their expression levels with clinicopathologic parameters. The median age of the patient cohort was 52.5 years (range, 30–86 years). Both TAP1 and TAP2 immunohistochemical expression levels correlated significantly with breast cancer characteristics (P < .001). TAP1 expression levels were low to negative in stage I breast tumors. TAP1 and TAP2 levels were significantly higher in grade 3 tumors than low-grade (grade 1 and 2) tumors. TAP1 and TAP2 expression levels were not significantly different among different levels of HER2-expressing tumors and did not vary by estrogen and progesterone receptor status or patient age. Both TAP1 and TAP2 overexpression in breast cancer might be an indicator of an aggressive breast tumor.

## Introduction

The transporter associated with antigen processing (TAP) is essential for peptide delivery from the cytosol to the lumen of the endoplasmic reticulum in the major histocompatibility complex (MHC) class I antigen presenting pathway. In the ER lumen, the delivered peptides are loaded on MHC class I molecules [1], where peptide-MHC class I complexes are targeted to the cell surface for antigen presentation to cytotoxic T cells. TAP protein−dependent inhibition of antigen presentation has evolved in human genetic diseases [2], viral infections [3–6], and in various human tumors [7–14]. Typically, expression of the TAP1 and TAP2 genes (TAP1/2) is downregulated in these diseases, or a mutation is present in one or both genes that hinder the peptide transport function of the subunits [15]. Increased understanding of which types or subsets of tumors downregulate TAP expression, the mechanism for downregulation of these transporters, and how it affects antigen processing may help improve antitumor immune responses and immunotherapy strategies.

Tumors can escape the immune response by impairing the MHC class I antigen processing pathway. Specifically, tumors can use selective processes that alter the levels of TAP1 or TAP2 (or both) to decrease peptide delivery (and subsequent binding to MHC class I peptides), ultimately evading cytotoxic CD8^+^ T cell recognition [16]. For example, melanoma cells have reduced TAP1 mRNA levels, and expression cannot be restored with interferon (IFN)-γ treatment [15]. TAP genes have also been mutated in small-cell lung cancer, leading to tumor escape from immune recognition. The tumor cells express normal levels of TAP1, but a mutation disrupts the peptide transport process [8]. Moreover, transduction of TAP1 and TAP2 genes in renal cell carcinoma repaired the expression and functional defects of these genes in this cancer type, enhanced the levels of MHC class I peptides on the surface of these cancer cells, and resulted in increased tumor necrosis factor−α secretion by T cells recognizing these cancer cells [17].

TAP-specific therapies are being developed to treat defects in tumors. Patients with small-cell and non−small-cell lung carcinoma were found to have defects in numerous class I antigen processing proteins [17]. A mechanism for enhancing tumor immunogenicity was investigated by infecting TAP1-deficient mouse lung carcinoma cells with a nonreplicating adenovirus expressing the human TAP1 gene (AdhTAP1). AdhTAP1 viral infection resulted in increased MHC class I peptide expression, increased antigen presentation, and increased susceptibility to antigen-specific cytotoxic T lymphocytes [17]. Additionally, in vivo viral infection in mice bearing lung cancer showed reduced tumor growth, increased numbers of CD8^+^ and CD4^+^ T cells infiltrating the tumor, and increased mouse survival. Further investigations have provided evidence supporting the inclusion of TAP genes in cancer vaccines because the presence of transporter proteins increases antigen presentation in tumors and thus increases immune recognition of tumors [18–21].

Currently, very little is known about TAP gene expression in breast cancer and whether these genes are inactive or downregulated in the tumor cells [22, 23]. To address this issue, we used triplicate breast cancer specimens from 160 female patients, analyzed in a parallel fashion by using tissue microarrays (TMAs) [24–26], to determine whether TAP1 and TAP2 were downregulated in specific subsets of breast cancer or were associated with other clinicopathologic features of tumors. To our knowledge, few previous investigation of TAP1 and TAP2 levels have examined associations with breast cancers, and those studies analyzed a limited number of tumor tissue samples [22, 27]. Two earlier studies investigated only TAP1 and human leukocyte antigen (HLA) class I levels in metastatic breast cancer. The first study suggests that low or defective TAP1 or calnexin in primary breast cancers may be at higher risk for developing metastasis due to defects in T cell-based immunosurveillance, while the other study suggests higher incidence of antigenic loss of HLA class I in metastatic vs primary breast cancer [22, 28]. Our study is the first to utilize TMAs to examine TAP1 and TAP2 levels of 480 breast cancer specimens from 160 female patients with breast cancer. We assessed the association of TAP1 and TAP2 levels with tumor grade and other clinicopathologic features such as stage, HER2/neu status, and estrogen receptor (ER) and progesterone receptor (PR) expression levels in an attempt to identify the relationship between loss of expression and aggressive tumor behavior. The identification of more complete gene expression profiles of tumors will enhance the understanding of protein regulation or dysregulation in tumor cells, and may be used to determine prognostic information or to identify patient-specific immunotherapy regimens [29].

## Materials and methods

### TMA construction

Formalin-fixed, paraffin-embedded (FFPE) breast tumor specimens (N = 160; procured and archived from 1998–2006) were obtained from the Mayo Clinic Tissue Registry (Rochester, Minnesota) and were matched based on ER status, tumor size, nodal status, and patient age. Two TMAs were constructed using a Beecher ATA-27 automated TMA construction system (Beecher Instrument). Breast tumors were stained with hematoxylin and eosin and all paraffin-embedded blocks were first reviewed by a pathologist to select representative areas of invasive tumor to be cored. From each FFPE tissue block, 3 tissue biopsies, 0.6 mm in diameter and 2.8 mm in depth, were randomly placed approximately 1.3 mm apart onto TMAs according to National Cancer Institute−recommended guidelines. Each TMA contained cores from control tissue, including non-neoplastic human liver and placenta. The two TMAs consisted of triplicate tissue biopsies (n = 480) representing 160 patients. Several patients were omitted from the final analysis because of lack of research consent or missing clinical information. One hundred sixty patients were represented in the final study for data analysis. HER2/neu-positive cases were scored using the published American Society of Clinical Oncology (ASCO) and College of American Pathologists (CAP) guidelines [30]. The (ASCO/CAP) IHC score of HER2/neu protein expression is on a scale of 0 to 3+ and has generally been adopted widely [30]. A score of 0–1+ represents a negative result, a score of 2+ is considered an equivocal IHC result. A score or 3+ is considered positive. ER and PR expression were considered low if less than 10% in tumor nuclei, and were considered high if more than 10% of tumor nuclei. The Mayo Clinic Internal Review Board for Human Subjects Research approved this study. All patients consented to the use of their biopsied specimens for biomedical research.

### Immunohistochemistry

Tissue sections were deparaffinized in xylene, dipped in decreasing concentrations of ethyl alcohol, and then rehydrated in distilled water. Antigen retrieval for TAP1 and TAP2 was performed by placing slides in preheated ethylenediaminetetraacetic acid as the retrieval solution in a steamer at 98°C for 30 minutes. The staining procedure was carried out in the Dako Autostainer Plus (using Dako reagents) as follows: tissue sections were treated with peroxidase blocking reagent for 5 minutes, washed with 1× wash buffer, and treated with serum-free protein blocking buffer for 5 minutes. The primary antibodies for TAP1 and TAP2 (gifts from Dr. Soldano Ferrone, Pittsburgh, PA) were diluted 1:100 in a background-reducing antibody diluent (Cat. No. S3022; Dako) and incubated for 60 minutes at room temperature. After rinsing with wash buffer, sections were incubated in secondary antibody (Cat. No. K4061, m-EnVision + horseradish peroxidase; Dako) for 15 minutes. The high-sensitivity diaminobenzidine chromogenic substrate system (Dako) was used for colorimetric visualization. Counter staining with hematoxylin followed by dehydration in increasing concentrations of ethyl alcohol and xylene were performed before a permanent coverslip was placed.

### Scoring

TMA scoring was performed by a board-certified, experienced pathologist (A.N.) who was masked to the clinicopathologic characteristics of the tumors. An Olympus BX51 upright light microscope was used to score cores at a magnification of 40×. TAP1 and TAP2 antibody staining of cores containing tumor were assessed using a quick-score method [31]. Cores were scored on a 0–3 scale on the basis of staining intensity (no staining = 0; light staining = 1; moderate staining = 2; strong staining = 3). The proportion of cells staining was also recorded as a percentage. Percentages were later converted to a score on a 1–6 scale (0%-4% = 1; 5%-20% = 2; 21%-40% = 3; 41%-60% = 4; 61%-80% = 5; 81%-100% = 6). The 2 scores were multiplied to obtain the final score, which could range from 0–18 (Table 1). The maximum quick score from each patient’s triplicate specimens was used for analysis. Cores from normal liver and placenta were used as negative controls.

**Table 1:** Quick score method accounting for the percentage of cells staining positive for a particular marker and the intensity of antibody staining.

Score (A)	Percentage of cells	Score (B)	Staining Intensity
1	0%-4%	0	No Staining (-ve)
2	5%-20%	1	Light
3	21%-40%	2	Moderate
4	41%-60%	3	Strong
5	61%-80%	**Quick Score = A X B** **Results (0–18)**	
6	81%-100%		

### Statistical analysis

All analyses were performed using JMP8 software (SAS Institute Inc). Linear regression analysis was performed to compare TAP1 vs TAP2 levels. One-way analysis of variance Newman-Keuls multiple comparison tests were performed to determine differences in TAP1 or TAP2 levels among specimens with different clinicopathologic characteristics. An unpaired Student *t* test was used for some analyses. *P* values < .05 were considered significant in all analyses. Several scores were grouped for analysis. TAP1 and TAP2 scores were divided into 3 groups (0–6 = negative/low; 7–12 = moderate; 13–18 = high) for proportion analysis against clinicopathologic characteristics. Fisher’s Exact test was used to determine statistical differences between proportions. ER and PR scores were categorized into 3 groups (0 = none; ≤10 = low; >10 = high) for analysis.

## Results

### Patient profiles

This study utilized breast cancer specimens obtained from 160 female patients during surgery. The median patient age was 52.5 years (range, 30–86 years) (Table 2). The majority of patients (83%) had a diagnosis of invasive ductal carcinoma at the time of surgery. For the remaining 17%, diagnoses were invasive lobular carcinoma, a combination of invasive ductal and lobular carcinoma, or other breast carcinomas. Seventy-eight tumors were clinically positive for HER2/neu and 81 were negative. HER2/neu status was not available for 1 patient. The tumors encompassed a broad range of stages and grades. Twenty-seven patients expressed high levels of ER, 65 patients expressed low levels, and 33 patients did not express ER. Twenty-one patients expressed high levels of PR, 68 patients expressed low levels, and 36 patients did not express PR. ER and PR information was incomplete for 35 patients.

**Table 2:** Clinical and pathologic characteristics (N = 160).

Characteristic	Value^a^
Age at surgery, median (range), y	52.5 (30–86)
Diagnosis (n = 159)	
Invasive ductal	132 (83.0)
Invasive ductal/lobular	9 (5.7)
Invasive lobular	9 (5.7)
Other	9 (5.7)
HER2/neu status (n = 159)	
0	47 (29.6)
1	34 (21.4)
2	7 (4.4)
3	71 (44.7)
Stage (n = 152)	
1	42 (27.6)
2	71 (46.7)
3	39 (25.7)
Grade (n = 125)	
1	4 (3.2)
2	63 (50.4)
3	58 (46.4)
Estrogen receptor status (n = 125)	
Negative	33 (26.4)
Low	65 (52.0)
High	27 (21.6)
Progesterone receptor status (n = 125)	
Negative	36 (28.8)
Low	68 (54.4)
High	21 (16.8)
Age	
≤55 y	88 (55.0)
>55 y	72 (45.0)

^a^ Data are shown as No. (%) unless otherwise indicated.

### Distributions of TAP1 and TAP2 expression

**Fig 1:**
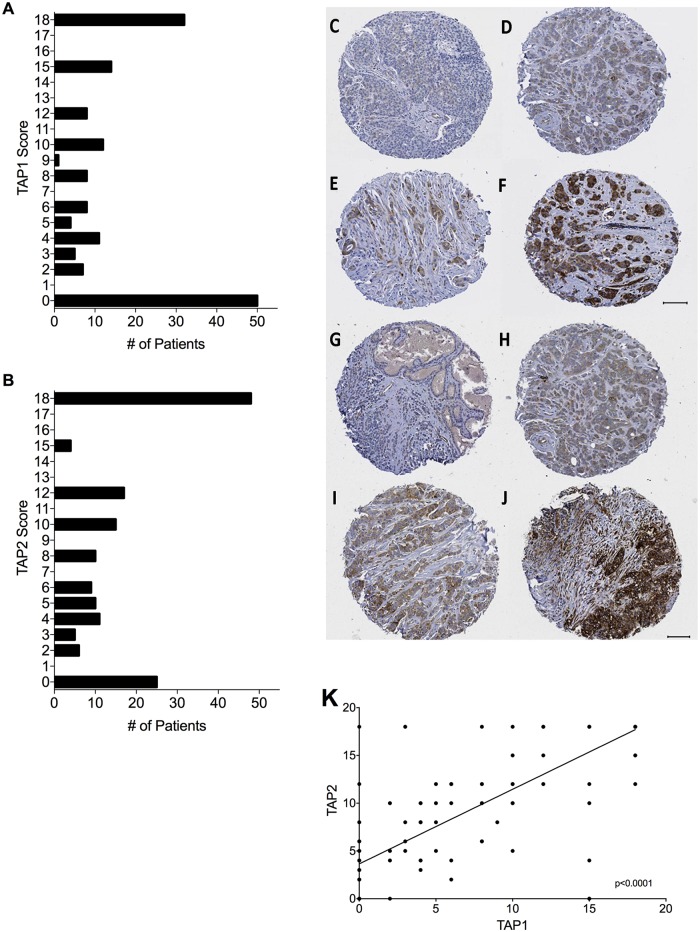
TAP1 and TAP2 expression in breast cancer specimens distributed across two tissue microarrays. TMAs were scored by a certified pathologist for intensity and extent of staining. (A and B) Distribution of maximum TAP1 and TAP2 quick scores, respectively, among individual breast cancer tumor specimens. (C-F) TAP1-stained TMA cores from breast tumor specimens. (C) Grade 3 invasive ductal carcinoma: 0 intensity × 0% extent. (D) Grade 3 invasive ductal carcinoma: 1 intensity × 80% extent. (E) Grade 2 invasive ductal carcinoma: 2 intensity × 60% extent. (F) Grade 3 invasive ductal carcinoma: 3 intensity × 90% extent. (G-J) represents TAP2-stained TMA cores from breast tumor specimens. (G) Grade 2 invasive lobular carcinoma: 0 intensity × 0% extent. (H) Grade 3 invasive ductal carcinoma: 1 intensity × 80% extent. (I) Grade 3 invasive ductal carcinoma: 2 intensity × 90% extent. (J) Grade 3 invasive ductal carcinoma: 3 intensity × 100% extent. TMA indicates tissue microarray. The scale bar in each image denotes 100 μm. (K) Both TAP1 and TAP 2 maximum expression levels were positively correlated by linear regression analysis (R^2^ = 0.6684; *P* < .001). TAP1 and TAP2 scores, determined by the quick-score method, ranged from 0 to 18.

The tumor specimens represented in the TMAs expressed the TAP1 and TAP2 subunits at various levels (Fig 1A–1J). The median quick score (staining × intensity score) was 6 (mean score, 7.6) for TAP1 and 10 (mean score, 9.6) for TAP2. Because TAP1 and TAP2 are both located on chromosome 6 and are under the control of IFN-γ to form a heterodimer in the endoplasmic reticulum membrane, we asked whether the expression of these subunits correlated with each other in the breast cancer specimens. The expression levels of TAP1 and TAP2, as measured by the quick score, were positively correlated in breast cancer specimens (Fig 1K). Linear regression analysis showed that the correlation was significant (R^2^ = 0.6684; *P* < .001).

### Stage, grade, HER2/neu, ER, and PR vs TAP1 and TAP2 levels

**Fig 2:**
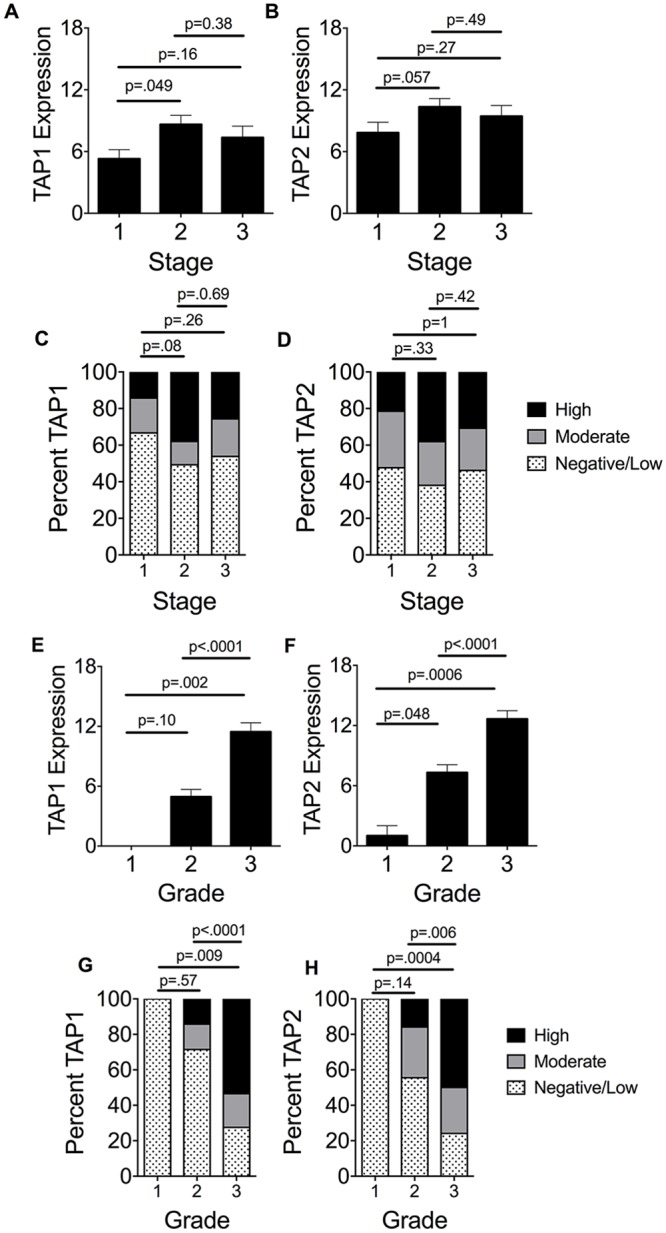
Correlation of expression levels of TAP1 and TAP2 in patients with stage and grade. (A and B) Mean ± SEM TAP1 and TAP2 expression levels, respectively, determined by the quick-score method, are shown; scores ranged from 0 to 18. Eight patients were excluded from this analysis because of missing stage data. (C and D) Percent of samples staining for TAP1 and TAP2 staining, respectively, among the 3 stage subsets. (E and F) Mean ± SEM TAP1 and TAP2 expression levels, respectively, determined by the quick-score method, are shown; scores ranged from 0 to 18. Thirty-five patients were excluded from this analysis because of missing grade data. The grade 1 subset had only 4 patients. (G and H) Percent of samples staining for TAP1 (G) and TAP2 (H) among the 3 grade subsets. Scores were divided into 3 groups (0–6 = negative/low; 7–12 = moderate; 13–18 = high). P values were calculated using unpaired Student’s T test for panels A, B, E, and F and The Fisher’s exact test for panels C, D, G, and H.

Because a previous group reported that TAP1 and TAP2 were downregulated in a limited number of high-grade breast cancer lesions [27], we sought to determine 1) whether TAP1 and TAP2 were downregulated in a large cohort of breast cancer patients and 2) whether downregulation was specific for certain clinicopathologic subsets in this cohort of patients. The mean levels of TAP1, but not TAP2 levels, differed slightly among patients with stage 1 and 2 disease (Fig 2A and 2B). However, the fraction of stage 1 tumors that were negative for TAP1 expression or expressed only low levels of TAP1 was not different than stage 2 tumors; similarly neither was that for TAP2 (Fig 2C and 2D). No statistically significant differences were detected in TAP1 and TAP2 expression among stage 2 and 3 tumors (Fig 2A–2D).

The mean TAP1 and TAP2 levels were significantly higher in grade 3 tumors than grade 1 and 2 tumors (Fig 2E and 2F). Most grade 1 and 2 tumors expressed TAP1 and TAP2 at low to negative levels, whereas most grade 3 tumors expressed TAP1 and TAP2 at high levels (Fig 2G and 2H). Statistically significant differences were detected between grade 1 and grade 2 or 3 tumors (grade 1 tumors were negative or low for TAP1 and TAP2, whereas grades 2 and 3 tumors expressed higher levels of these subunits).

**Fig 3:**
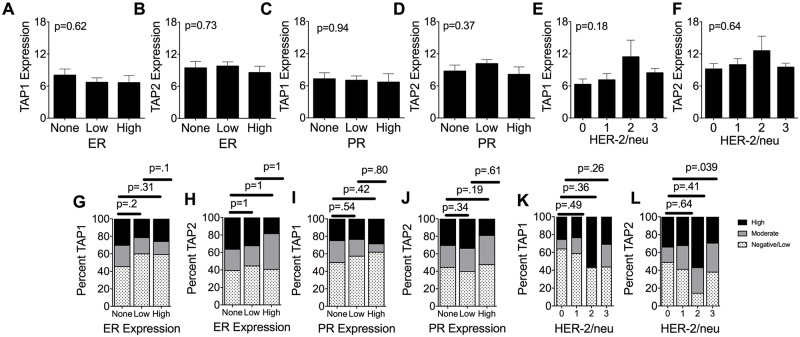
Correlation of expression levels of TAP1 and TAP2 in patients categorized based on progesterone receptor (PR) status, estrogen receptor (ER) status, and HER2/neu expression levels. (A and B) Expression levels of TAP1 (A) and TAP2 (B) in patients with breast cancer patients expressing negative, low, or high levels of estrogen receptors (ERs). Thirty-five patients were excluded from the analysis because of missing ER expression data. (C and D) Expression levels of TAP1(C) and TAP2 (D) in patients with breast cancer expressing negative, low, or high levels of progesterone receptors (PRs). (E and F) Expression levels of TAP1(E) and TAP2 (F) in patients with breast cancer expressing negative, low, or high levels of progesterone receptors (PRs). Thirty-five patients were excluded from the analysis because of missing PR expression data. (G and H) Percent of samples staining for TAP1 (G) and TAP2 (H) among the 3 ER expression groups. (I and J) Percent of different TAP1 (I) and TAP2 (J) staining levels among the 3 PR expression groups. (K and L) Percent of samples staining for TAP1(K) and TAP2 (L) among the 4 HER2/neu expression groups. Expression levels of TAP1 and TAP2 in patients negative and positive for HER2/neu breast cancer TAP1 and TAP2 scores were divided into 3 groups (0–6 = negative/low; 7–12 = moderate; 13–18 = high) for panels A-F. P values were calculated using unpaired Student’s T test for panels A-F and The Fisher’s exact test for panels G-L.

Mean TAP1 and TAP2 levels were also compared to the levels of ER (Fig 3A and 3B), PR (Fig 3C and 3D), and HER-2/neu (Fig 3E and 3F). In all analyses, no significant differences were detected in the TAP1 and TAP2 staining levels among the various levels of the clinicopathologic features. Similarly, the proportions of patient samples expressing ER (Fig 3G and 3H), PR (Fig 3I and 3J), and HER-2/neu (Fig 3K and 3L) were not statistically different with the one exception being that the fraction of HER-2/neu 3+ tumors expressed significantly more moderate to high levels of TAP2. Overall there is a trend toward both higher TAP1 and TAP2 levels among the higher HER-2/neu categories (2 and 3+), however this did not reach statistical significance, likely due to the small number of patients represented in the HER-2/neu 2 category (n = 7, Table 2).

## Discussion

The present study investigated the levels of TAP1 and TAP2 in 480 breast cancer specimens representing 160 patients with breast cancer. We showed that higher levels of TAP1 and TAP2 were present in high-grade breast carcinoma and that TAP1 was expressed at higher levels in stage 2 tumors than in stage 1 tumors. The results of this study contradict a previously published report investigating TAP1 and TAP2 levels in 53 patients with breast carcinoma [27], as well as a report investigating TAP1 levels in primary vs metastatic breast cancer [28]. Vitale et al [27] found that TAP1 and TAP2 downregulation occurred in 68% of high-grade breast cancer lesions and in none of the low-grade lesions. Additionally, they reported that HLA class I antigen was also downregulated and that this change was associated with TAP downregulation. Downregulation of TAP is required for cytotoxic T-lymphocyte recognition [16]. Kaklamanis et al [28] measured TAP1 levels in metastatic breast cancer (a subgroup not investigated in our study) and discovered that TAP1 loss was associated with total loss of HLA class I in primary and metastatic tumors. In our study and that of Vitale et al [27], both FFPE tissue sections and identical anti-TAP1 and TAP2 antibodies [31] were used, so the differences likely are not due to differences in methodology.

Many investigations in the literature have demonstrated loss of TAP1 and TAP2 expression in human cancers [7–14, 31–35]. These previous studies suggest that class I antigen processing defects may be associated with malignant transformation of cells. Similarly, Liu et al reported lower expression levels of TAP1 (p = 0.006) and TAP2 (p = .0004) in patients with more advanced stage breast cancer [22]. Our study has shown the opposite, with TAP1 and TAP2 being expressed at lower levels in early stages of breast cancer. Our results also indicate that as the tumors become less differentiated (higher grade), they express higher levels of TAP1 and TAP2. One potential explanation for these results is that more immune infiltrates may be present in the tumor microenvironment in advanced stage and higher grade tumors. These immune cells may be secreting IFN-γ, thus causing upregulation of the TAP subunits in the tumor cells [13, 36, 37]. Alternatively, as the tumor cells lose control of their cell cycle and become less differentiated, they may lose the ability to control expression of the *TAP* genes. Although the field of immunology and immunotherapy has always emphasized the tumor’s ability to downregulate critical players in the class I antigen processing pathway, our results suggest that tumors may not be able to effectively downregulate *TAP* genes at advanced stages. Liu et al has shown that the extent of CD8^+^ T-cell infiltration in the primary site of breast cancer was positively associated with expression levels of TAP1 (*P*≤.004) [22]. High levels of TAP1 positively correlated with longer relapse-free survival in patients with breast cancer who underwent chemotherapy [23].

One of the limitations of the current study is the small representation of grade 1 tumors (only 4 patients had grade 1 tumors), so the study underpowered to draw conclusions about those subsets of patients.

Because prior evidence in the literature suggested loss of TAP expression in various cancers, TAP has emerged as a strong candidate for adjuvant immunotherapies and vaccines aimed at increasing the number of peptide antigens that tumors present to CD8^+^ cytotoxic T cells [18, 38–40]. Our results indicate that these strategies may not be necessary in advanced stages and higher grades of breast cancer, which already display the TAP1 and TAP2 subunits. These treatment strategies may need to be carefully tailored to specific cancer types and subgroups of patients to have maximal benefit.
